# Islet cell replacement and transplantation immunology in a mouse strain with inducible diabetes

**DOI:** 10.1038/s41598-022-13087-3

**Published:** 2022-05-31

**Authors:** Preksha Bhagchandani, Charles A. Chang, Weichen Zhao, Luiza Ghila, Pedro L. Herrera, Simona Chera, Seung K. Kim

**Affiliations:** 1 Department of Developmental Biology, Stanford University School of Medicine, Stanford, CA 94305 USA; 2 Department of Clinical Science, University of Bergen, Bergen, Norway; 3 Department of Genetic Medicine and Development, University of Geneva, Geneva, Switzerland; 4 Department of Medicine (Endocrinology Division), Stanford University School of Medicine, Stanford, CA 94305 USA; 5 Department of Pediatrics (Endocrinology Division), Stanford University School of Medicine, Stanford, CA 94305 USA; 6 Stanford Diabetes Research Center, Stanford University School of Medicine, Stanford, CA 94305 USA; 7 JDRF Center of Excellence, Stanford University School of Medicine, Stanford, CA 94305 USA

**Keywords:** Endocrine system and metabolic diseases, Transplant immunology, Biological techniques, Immunology, Stem cells, Diseases, Endocrinology

## Abstract

Improved models of experimental diabetes are needed to develop cell therapies for diabetes. Here, we introduce the B6 RIP-DTR mouse, a model of experimental diabetes in fully immunocompetent animals. These inbred mice harbor the H2^b^ major histocompatibility complex (MHC), selectively express high affinity human diphtheria toxin receptor (DTR) in islet β-cells, and are homozygous for the *Ptprc*^*a*^ (CD45.1) allele rather than wild-type *Ptprc*^*b*^ (CD45.2). 100% of B6 RIP-DTR mice rapidly became diabetic after a single dose of diphtheria toxin, and this was reversed indefinitely after transplantation with islets from congenic C57BL/6 mice. By contrast, MHC-mismatched islets were rapidly rejected, and this allotransplant response was readily monitored via blood glucose and graft histology. In peripheral blood of B6 RIP-DTR with mixed hematopoietic chimerism, CD45.2 BALB/c donor blood immune cells were readily distinguished from host CD45.1 cells by flow cytometry. Reliable diabetes induction and other properties in B6 RIP-DTR mice provide an important new tool to advance transplant-based studies of islet replacement and immunomodulation to treat diabetes.

## Introduction

Studies to advance islet transplantation in mice would benefit from newer models of experimental diabetes^1^. The most widely-used models are those involving administration of islet β cell toxins, like streptozotocin (STZ)^2^. The simplicity of conditional diabetes induction with this drug is offset by multiple disadvantages, including extra-islet toxicity and immuno-modulatory effects of STZ, inconsistent β cell ablation, and variable diabetes induction^3,4^. Thus, studies of cell-based therapies for diabetes, including those aiming to achieve islet transplant tolerance, would benefit from fully immunocompetent mouse models that address these concerns^5,6^.

In prior studies, we generated mice permitting genetically-encoded β cell ablation and highly penetrant diabetes induction^7^. Pancreatic islet β cells in these mice express the membrane-anchored form of human heparin binding EGF-like growth factor (HB-EGF), which binds diphtheria toxin (hereafter, ‘diphtheria toxin receptor’ or DTR). Upon diphtheria toxin (DT) administration and binding to DTR, β cell protein synthesis is suppressed from inhibition of elongation factor 2 (EF2), an essential factor for ribosomal translocation, leading to cell death^8^. Selective HB-EGF expression in β cells is directed from an insulin gene promoter (RIP) element^7^. Since wild-type murine cells do not bind DT, DTR-mediated cell ablation is a highly sensitive and specific method that allows targeting of β cells in RIP-DTR mice without off-target host cell damage or toxicity that impacts metabolism or immune function^9^. In RIP-DTR adult mice, DT injection results in > 99% β cell ablation, with low overall β cell regeneration rate thereafter^7^. Although available RIP-DTR mice have superior characteristics of diabetes induction efficiency and specificity as compared to STZ challenge^10^, they are on mixed or immunocompromised genetic backgrounds, and are therefore poorly suited for transplantation tolerance studies. In addition, allotolerance studies involving mixed chimerism or cell-based therapies in the setting of diabetes would benefit from methods to distinguish donor and host cells, and to readily assess tolerance status of transplanted islets.

Here, we detail development and characterization of a genetically inbred, immunocompetent mouse strain harboring the RIP-DTR transgene, which permits diabetes induction with 100% penetrance after DTR-mediated β cell ablation. We incorporated the mutant CD45.1 cell surface marker expressed on hematopoietic cells to facilitate distinction of host and donor cells, and demonstrated multiple uses of this strain for islet transplantation studies. This strain is hereafter called “B6 RIP-DTR.” Our study describes uses of this improved diabetes model to advance islet transplantation and tolerance studies in overtly diabetic mice.

## Results

### Generation, genetics, and immunological phenotypes of B6 RIP-DTR mice

RIP-DTR mouse strains were initially generated and maintained on mixed B6/CBA genetic backgrounds for studies of beta cell regeneration^7,10,11^. To make an inbred strain of RIP-DTR mice for transplantation studies, we repeatedly backcrossed to B6.SJL-*Ptprc*^*a*^* Pepc*^*b*^/BoyJ mice, commonly referred to as Pep Boy or B6 CD45.1 (hereafter, B6 CD45.1), to generate an inbred line with a defined histocompatibility genotype. After backcrossing, we analyzed B6 CD45.1 RIP-DTR mice (abbreviated B6 RIP-DTR) using single nucleotide polymorphism (SNP) genome scanning analysis (Supplementary Table S1) to measure relative C57BL/6J identity. After four rounds of backcrossing to B6 CD45.1 and subsequent sibling matings to maintain homozygosity for RIP-DTR (N4F4 breeding scheme; “Methods”), we achieved an average 96% C57BL/6J identity at 120 single nucleotide polymorphisms (SNPs) analyzed across 20 chromosomes from 12 mice. It is not possible to obtain 100% C57BL/6J identity throughout the genome, due to the presence of multiple polymorphisms between the B6 CD45.1 mice used for backcrossing and C57BL/6J strain, most notably on chromosome 1^12^. We achieved 98% C57BL/6J identity, if chromosome 1 is excluded from analysis. B6 RIP-DTR have multiple strain characteristics similar to the commonly used C57BL/6J mouse strain. B6 RIP-DTR mice have lifespans of at least one year, and average litter size of 7 ± 2 (n = 9). At 10 weeks of age, average male weight is 27.3 g ± 2.3 (n = 13), and average female weight is 19.7 g ± 1.5 (n = 13), similar to C57BL/6J mice^13^. In sum, our genotyping indicated successful generation of inbred B6 RIP-DTR mice.

B6 RIP-DTR mice exhibit 100% C57BL/6J identity on chromosome 17 (Supplementary Table S1). Chromosome 17 contains all class I and class II major histocompatibility complex (MHC) genes responsible for antigen presentation and distinguishing self from non-self^14^, which are inherited together through linkage^15^. To phenotype the MHC haplotypes of B6 RIP-DTR mice, we used flow cytometry to analyze peripheral blood lymphocytes (Supplementary Fig. S1, Supplementary Table S2). The *MHC Class II* gene I-A is expressed on antigen-presenting cells, and the I-A^b^ allele is expressed by C57BL/6J mice, while the I-A^k^ allele is expressed by CBA/J mice. I-A^b^ only was expressed by blood cells in both C57BL/6J and B6 RIP-DTR mice. I-E^k^, another *MHC Class II* gene expressed by CBA/J blood cells, was not detectable in C57BL/6J nor B6 RIP-DTR mice. The *MHC Class I* gene H-2K is expressed ubiquitously, and the H-2K^b^ allele is characteristic of C57BL/6J mice while H-2K^k^ is characteristic of CBA/J mice. Flow cytometry revealed that only H-2K^b^ was expressed by blood cells in both C57BL/6J and B6 RIP-DTR mice. Thus, molecular phenotyping confirmed that B6 RIP-DTR mice express *MHC Class I* and *Class II* gene products characteristic of C57BL/6J mice with the MHC haplotype H2^b^.

### RIP-DTR and CD45.1 in B6 RIP-DTR mice

We verified the presence of the RIP-DTR transgene in B6 RIP-DTR mice using PCR (Supplementary Fig. S2; “Methods”). Since this transgene was inserted in the *Hprt* locus of the X chromosome, female RIP-DTR heterozygotes remain normoglycemic despite 50% beta cell loss after diphtheria toxin administration, reflecting random X chromosome inactivation in β cells^7^. Therefore, we sought to produce female B6 RIP-DTR homozygous for RIP-DTR, and males hemizygous for RIP-DTR. To achieve this, we developed a comparative qPCR method to distinguish female heterozygotes from homozygotes (Supplementary Fig. S2). Homozygous females (2 copies of RIP-DTR) result in a one cycle threshold (CT) difference from heterozygous females with one copy of RIP-DTR. Thus, our methods reliably measured RIP-DTR copy number, and generated breeder pairs that ensure production of B6 RIP-DTR female mice homozygous for RIP-DTR and males hemizygous for RIP-DTR.

**Figure 1:**
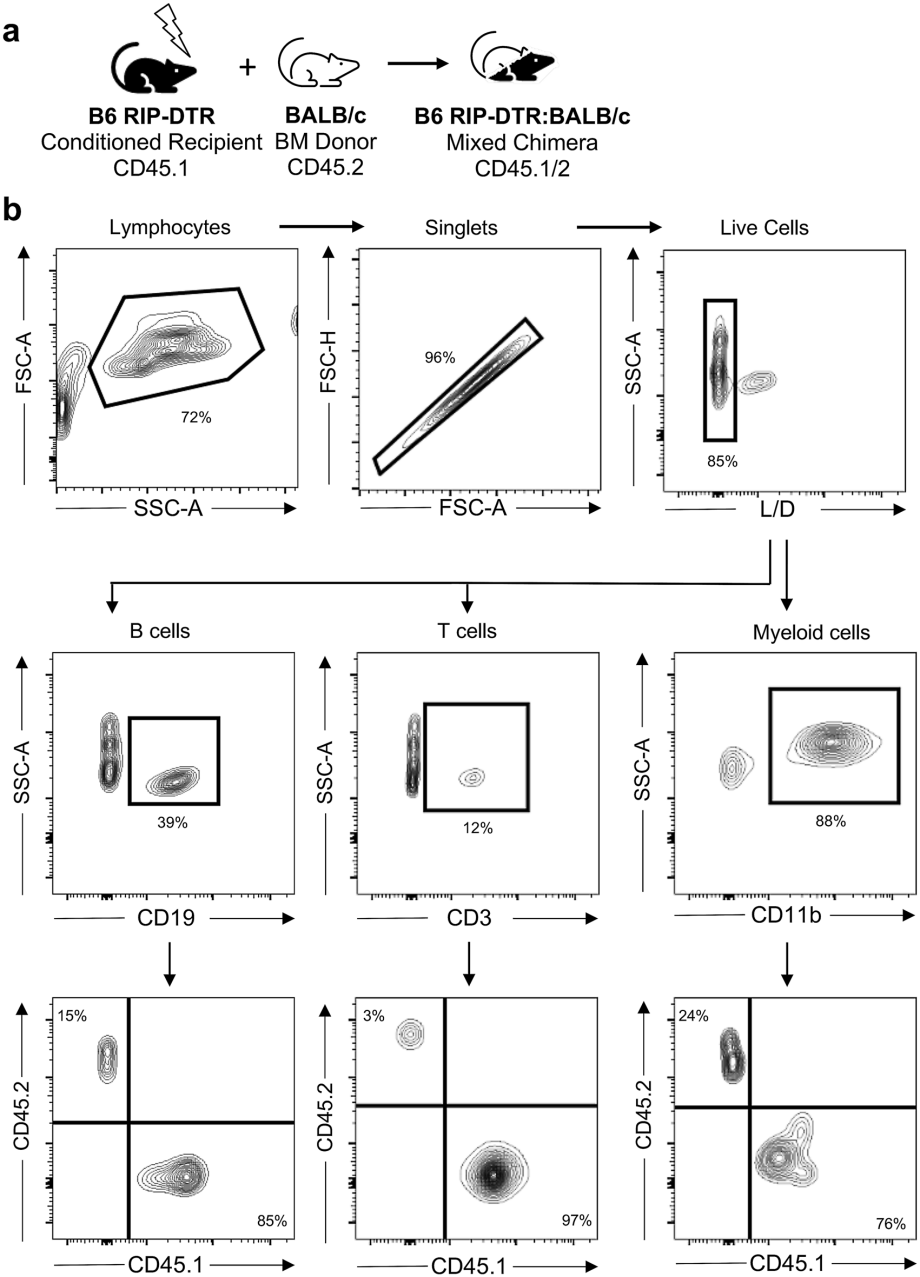
Phenotyping of CD45 in B6 RIP-DTR mice with BALB/c mixed chimerism. (**a**) Schematic showing generation of mixed chimerism in B6 RIP-DTR mice (CD45.1) using bone marrow (BM) from BALB/c CD45.2 donors. (**b**) Representative flow analysis of peripheral blood from a B6 RIP-DTR mixed chimera at 8 weeks after hematopoietic cell transplant with 1.5 × 10^6^ CD45.2 BALB/c donor hematopoietic cells. Live single cells are gated on CD19 to distinguish B cells, CD3 to distinguish T cells, or CD11b to distinguish myeloid cells, which are subsequently gated on CD45.1 and CD45.2 to distinguish host and donor cells.

To facilitate studies in B6 RIP-DTR mice receiving allogeneic hematopoietic cell transplantation, we sought to make immune cells in B6 RIP-DTR mice readily distinguishable from allogeneic donors. Most available wild-type mouse strains express the CD45.2 epitope on immune cells, encoded by the Ptprc^b^ allele. Thus, our breeding strategy aimed to produce B6 RIP-DTR homozygous for the mutant Ptprc^a^ allele, which encodes CD45.1, an isoform of the CD45 cell surface protein readily distinguishable from CD45.2 using flow cytometry^4^. To achieve this goal, we backcrossed RIP-DTR mice with B6 CD45.1 mice. We generated genotyping tools (“Methods”) to track and confirm homozygosity of the Ptprc^a^ allele. Specifically, dual endpoint qPCR was used to distinguish the Ptprc^a^ allele from Ptprc^b^ in the initial backcrosses, and in all subsequent generations of B6 RIP-DTR CD45.1 mice (Supplementary Fig. S2). Ptprc^a^ genotyping was confirmed by flow cytometry analysis on peripheral blood showing CD45.1 expression only (Supplementary Fig. S3). To demonstrate additional advantages of this novel strain for studies that require differentiation of host and donor hematopoietic cells, we established mixed hematopoietic chimeras using B6 RIP-DTR host mice and BALB/c bone marrow donors (Fig. 1a, “Methods”). In stable mixed chimeric B6 RIP-DTR mice, host CD45.1^+^ immune cells, including CD19^+^ B cells, CD3^+^ T cells, and CD11b^+^ myeloid cells, were readily distinguished from CD45.2^+^ donor cells in peripheral blood by flow cytometry (Fig. 1b). Thus, our approaches generated and validated uses of inbred B6 RIP-DTR mice homozygous for the CD45.1 cell surface marker.

### Fully penetrant diabetes in RIP-DTR mice after diphtheria toxin injection

**Figure 2:**
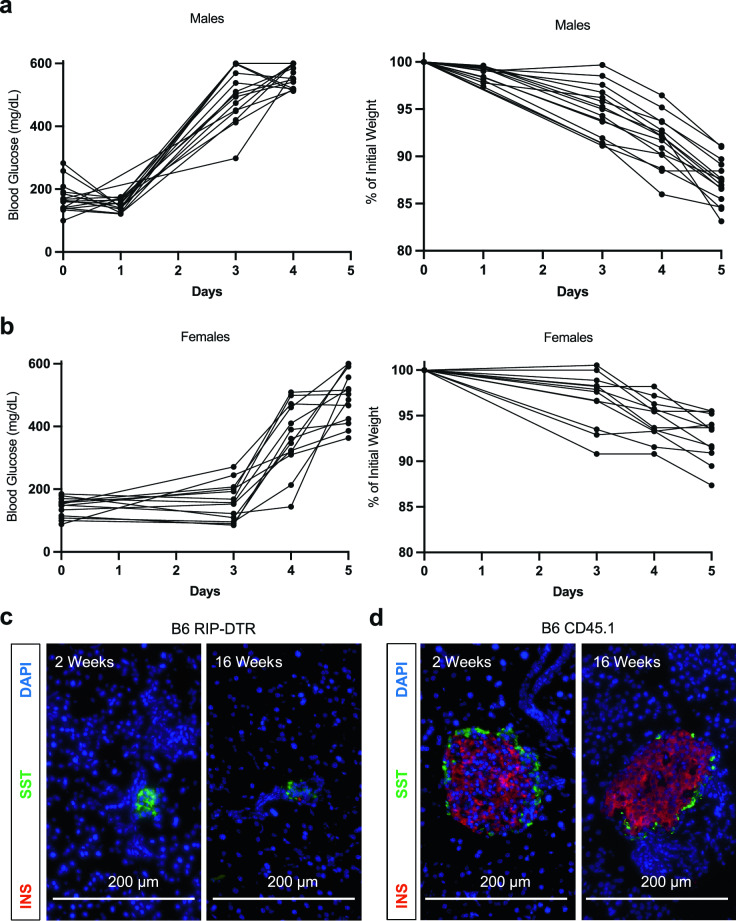
Diabetes in RIP-DTR mice after DT administration. (**a**) Non-fasting blood glucose and percentage of starting weight in RIP-DTR mice after single dose administration of DT (i.p.) in n = 16 hemizygous males and (**b**) n = 13 homozygous females. DT was injected on day 0 after baseline non-fasting blood glucose was recorded. Mice were between 8 and 24 weeks of age at time of injection, and no exogenous insulin was administered. (**c**) Representative histology of pancreas taken at 2 weeks and 4 months from B6 RIP-DTR or (**d**) B6 CD45.1 mice given single dose of DT. After confirming hyperglycemia on two consecutive days, we maintained the health of diabetic B6 RIP-DTR mice up to the 2-week timepoint by providing 40 U/kg exogeneous insulin daily. To maintain diabetic B6 RIP-DTR mice to 4 months, insulin pellets (“Methods”) were administered subcutaneously after confirmation of hyperglycemia on two consecutive days. *INS* Insulin, *SST* somatostatin.

To measure the efficiency of diabetes induction in B6 RIP-DTR mice, we administered a single dose of diphtheria toxin (DT) intraperitoneally (i.p.) to males (n = 16) and females (n = 13) between 8 and 24 weeks of age (Fig. 2; “Methods”). Blood glucose was measured before and after injection until mice were overtly hyperglycemic (> 250 mg/dL). 100% of male mice were hyperglycemic approximately 3 days after DT injection, regardless of age (Fig. 2a). Likewise, 100% of female mice were also hyperglycemic by approximately 4 days after DT injection, regardless of age (Fig. 2b). Within 24–48 h after the onset of hyperglycemia, the health of diabetic mice deteriorated, reflected by rapid weight loss, unless provided exogenous insulin. The observed sex difference between male and females for time to diabetes onset could reflect differences in sex hormones, where estrogen confers mild protection from hyperglycemia^16,17^. For subsequent transplantation studies, we were able to consistently predict the timing of hyperglycemia onset for both sexes. Additionally, histology of the pancreas from RIP-DTR mice at 2 weeks and 4 months after single DT injection shows effective β cell ablation, with little to no insulin producing cells remaining (Fig. 2c). This is consistent with prior reports of extremely low β cell regeneration rate after DT-dependent ablation in RIP-DTR mice with a mixed CBA/B6 genetic background^7^. By contrast, histology of the pancreas from control B6 CD45.1 mice injected with DT revealed normal-appearing islets that included intact β cells and other islet endocrine cells (Fig. 2d). Thus, our studies revealed that the RIP-DTR transgene was functional in B6 RIP-DTR mice, and resulted in rapid and reliable β cell loss, and conditional diabetes induction.

### Islet transplantation in B6 RIP-DTR mice

**Figure 3:**
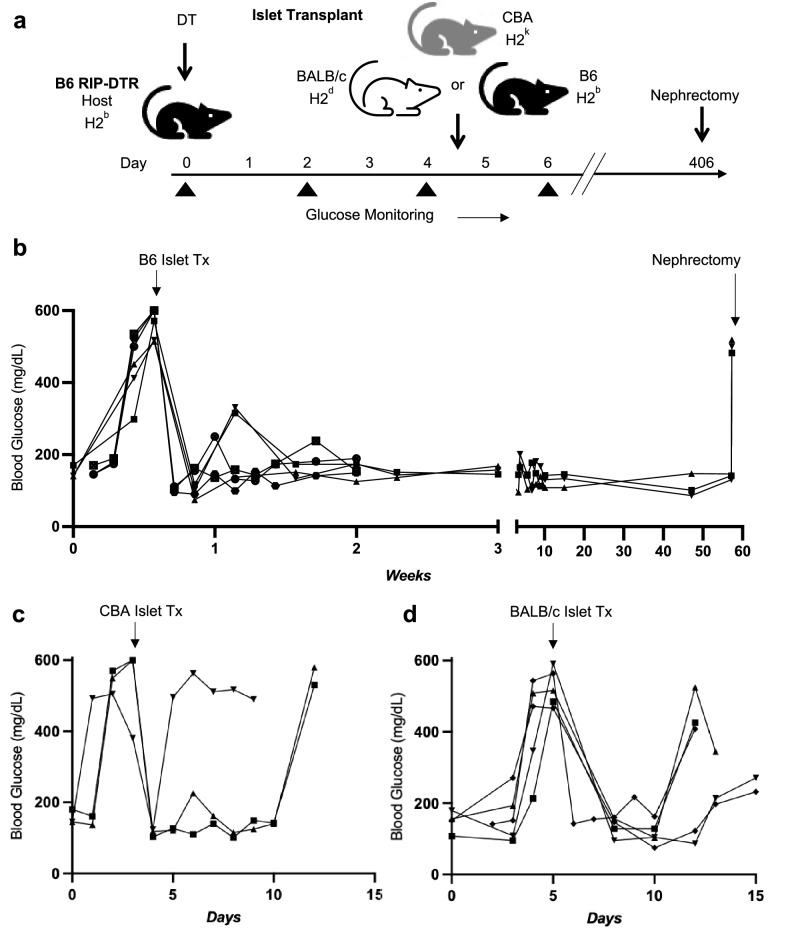
Islet transplantation in diabetic B6 RIP-DTR mice. (**a**) Schematic of congenic and allogeneic islet transplantation in diabetic B6 RIP-DTR mice. (**b**) Male RIP-DTR mice (n = 6; age 10–24 weeks) were injected with DT on day 0 and transplanted with C57BL/6J islets on day 4 after confirmation of hyperglycemia. Mice (n = 6) were monitored for about 2 weeks post-transplant. Mice (n = 3) were monitored for approximately another 1 year, until nephrectomy was performed at 56 weeks post-transplant to remove the islet graft. (**c**) Male RIP-DTR mice (n = 3; age 24 weeks) were injected with DT on day 0 and transplanted with allogeneic CBA/J islets on day 4 after confirmation of hyperglycemia. Mice were monitored for repeat hyperglycemia for up to 14 days. (**d**) Female and male RIP-DTR mice (n = 5; 4F, 1M, age 10–24 weeks) were injected with DT on day 0 if female and day 1 if male and transplanted with BALB/c (allogeneic) islets on day 5 after confirmation of hyperglycemia. Mice were monitored for repeat hyperglycemia for up to 14 days. In all cohorts, a single dose of exogenous insulin (40 U/kg) was administered on the morning of islet transplantation following confirmation of hyperglycemia. No further exogenous insulin treatment was provided.

**Figure 4:**
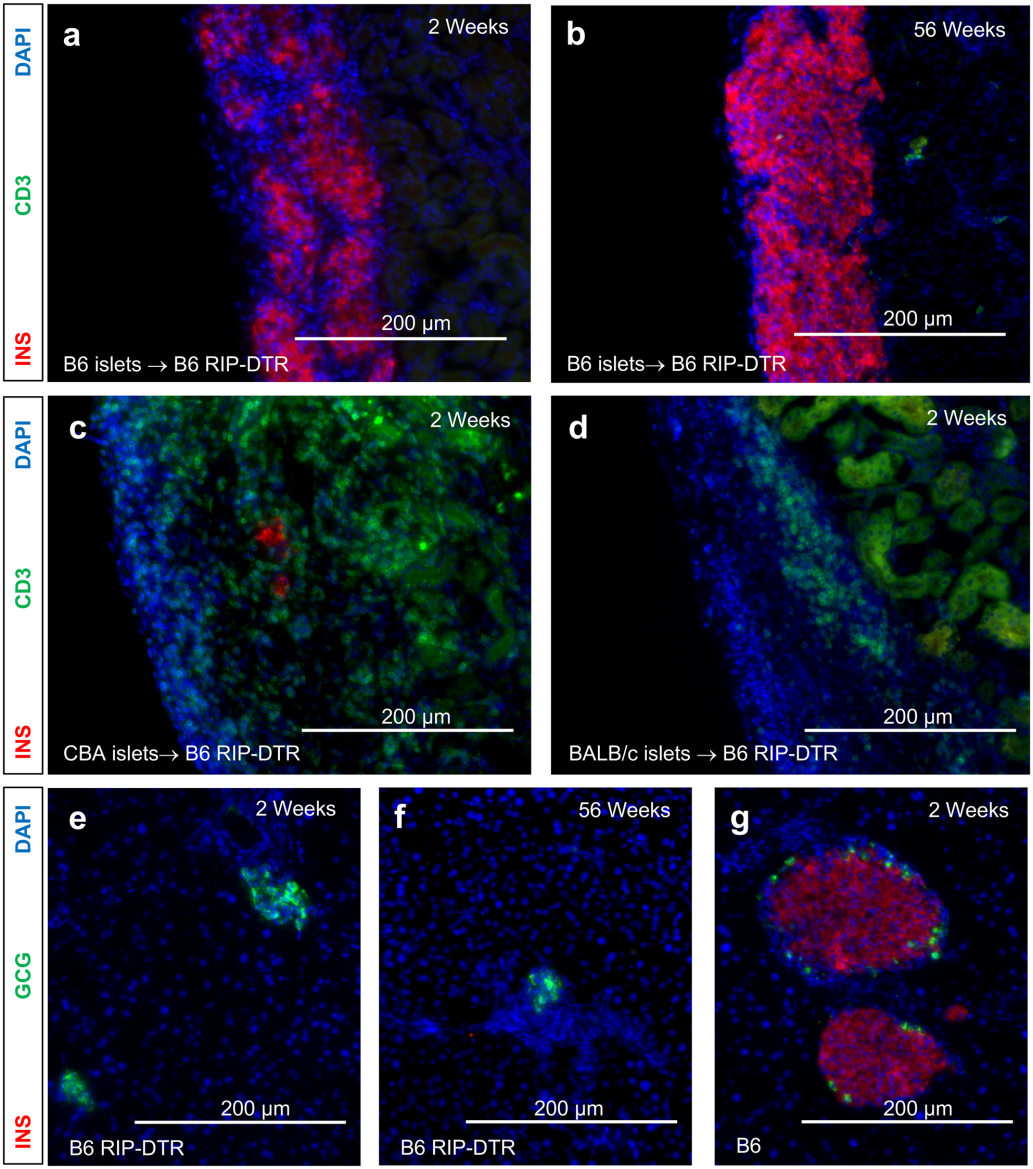
Histological assessment of transplanted and pancreatic islets. (**a**) Representative histology of C57BL/6J islet graft at 2 weeks and (**b**) 1-year post-transplant into B6 RIP-DTR mice. (**c**) Representative histology of CBA/J islet graft at approximately 2 weeks post-transplant into B6 RIP-DTR mice. (**d**) Representative histology of BALB/c islet graft at approximately 2 weeks post-transplant into B6 RIP-DTR mice. (**e**) Representative histology of B6 RIP-DTR pancreas from animals transplanted with C57BL/6J islets at 2 weeks and (**f**) 1 year after single DT injection. No exogenous daily insulin was necessary due to the function of transplanted islets. (**g**) Representative histology of B6 CD45.1 pancreas (wild type) at 2 weeks after single DT injection. *INS* Insulin, *CD3* CD3^+^ T cells, *GCG* glucagon.

To validate the use of B6 RIP-DTR mice for studies of immune tolerance, we transplanted diabetic mice with wild-type H2^b^ C57BL/6 islets, H2^k^ CBA/J islets, or MHC-mismatched H2^d^ BALB/c islets, and subsequently monitored blood glucose (Fig. 3a; Supplementary Table S2). Six B6 RIP-DTR mice, at 10–24 weeks of age, were injected with DT, and after hyperglycemia onset were transplanted in the renal sub-capsular space with islets from C57BL/6 donors (Fig. 3b). By 3 days after transplantation, all recipients became euglycemic without supplemental insulin. To confirm the functional status of the transplanted islets, grafts were removed from three mice at 10–12 days, and histology showed intact insulin^+^ β cells without CD3^+^ T cell infiltration and little CD45^+^ immune cell infiltrate (Fig. 4a, Supplementary Fig. S4). The remaining three mice remained euglycemic for at least 56 weeks following islet transplantation (Fig. 3b). Reversion to hyperglycemia invariably followed nephrectomy of the islet graft-bearing kidney at 56 weeks, confirming long-term tolerance of the C57BL/6 islet graft as expected. Furthermore, histology of islet grafts after 56 weeks showed intact insulin producing β cells without detectable CD3^+^ T cell infiltration and little CD45^+^ immune cell infiltrate (Fig. 4b; Supplementary Fig. S4). Pancreatic histology in B6 RIP-DTR mice at 2 weeks or 1-year after DT injection showed persistent β cell ablation, in contrast to healthy pancreas from DT-injected B6 CD45.1 mice (Fig. 4e–g). In summary, C57BL/6 islets are functional after transplantation and therefore durably tolerated as congenic in the novel B6 RIP-DTR mouse strain.

To verify that B6 RIP-DTR mice are no longer immunologically compatible with the prior mixed B6/CBA background, we transplanted CBA/J islets into three B6 RIP-DTR mice after administration of DT and confirmation of hyperglycemia (Fig. 3c). After transplantation and reversion to euglycemia, none of these mice remained euglycemic beyond two weeks, consistent with the timeline of adaptive immunological rejection. As expected, similar outcomes were observed after transplanting third-party MHC-mismatched BALB/c islets into diabetic B6 RIP-DTR (Fig. 3d). Histologic studies for both groups revealed heavy infiltration of the islet graft site with CD3^+^ and CD45^+^ immune cells, with few or no remaining insulin^+^ β cells (Fig. 4c,d; Supplementary Fig. S4). Thus, our breeding scheme successfully produced H2^b^ B6 RIP-DTR mice that are no longer compatible with H2^k^ donors and normally reject MHC-mismatched grafts.

## Discussion

Improved animal models of experimental diabetes are essential to advance and translate studies of islet transplantation tolerance and cell-based therapies in diabetes. Genetically-encoded DTR-mediated β cell ablation is reproducible, > 99% complete, and leads to diabetes with 100% penetrance^7,10^. However, genetically ambiguous or immunodeficient backgrounds of available RIP-DTR strains have precluded studies of allotolerance in islet transplantation. Here, we detail use of backcrossing, directed intercrossing, genotyping, and molecular phenotyping to produce a genetically inbred strain of B6 RIP-DTR mice with MHC haplotype H2^b^, and studies with this new strain that demonstrate effective β cell ablation and efficient diabetes induction. Diabetes reversal after transplantation with congenic B6 islets but not with MHC-mismatched CBA/J islets *functionally validates* the H2^b^ MHC haplotype of the B6 RIP-DTR strain, and supports the use of these mice in transplantation studies that require defined histocompatibility. Moreover, backcrosses to incorporate the CD45.1 hematopoietic cell marker aided flow cytometry-based studies of transplanted hematopoietic cell lineages^18^ in B6 RIP-DTR mice.

The RIP-DTR transgene has been used to induce β cell death, without immune infiltrates or autoimmunity^7^. Diphtheria toxin administration initiates cell lysis and intranucleosomal DNA fragmentation (apoptosis)^7,19^. Since murine cells lack the DT receptor, cells in RIP-DTR mouse strains engineered to express a human transgene encoding this receptor can be selectively destroyed, with minimal pancreatic immune infiltration. Although some leakiness of RIP has been reported in the central nervous system^20,21^, no distinguishable phenotypes, including metabolic or immune features, have been reported in RIP-DTR mice. To further mitigate such concerns, it may be possible to use formulations of diphtheria toxin that do not cross the blood brain barrier^22^. In contrast, widely-adopted chemotoxic methods to destroy β cells and induce diabetes in rodents, like STZ challenge^3,4,23^, are known to cause undesired off-target damage to cells in the liver, nervous system, cardiovascular system, respiratory system, kidneys, and reproductive system^4^. STZ challenge may also confound studies of transplantation tolerance, because of the association of STZ administration with decreased T cell numbers^24,25^. In addition to undesirable effects, previous studies directly comparing the STZ and RIP-DTR models demonstrated variable, lower efficiency of β cell ablation with STZ challenge^10^, and spontaneous recovery from STZ-induced diabetes^26^. Thus, while STZ can be used in a variety of strains, the variability of responses and efficiencies in different strains and sexes leads to dosing challenges^3,4,23^.

In summary, the unique combination of the CD45.1 hematopoietic cell marker and the *Ins2-HBEGF* allele in the B6 RIP-DTR strain provides a model of experimental diabetes that offers specificity, efficiency, and reproducibility beyond extant models, and is well-suited for a broad range of studies related to transplantation-based islet replacement. These include studies of islet transplantation tolerance, mixed chimerism, immunomodulation, and cell-based therapies in the setting of diabetes. These features should promote wider adoption of B6 RIP-DTR mice for studies of islet replacement and islet transplantation immunology.

## Materials and methods

### Animals

Female and male B6 (Stock # 000664), B6 CD45.1 (Stock #: 002014), BALB/c (Stock #: 000651), CBA (Stock #: 000656), and NSG (Stock #: 005557) mice were purchased from The Jackson Laboratory (Bar Harbor, ME). RIP-DTR mice were originally on a mixed B6/CBA genetic background (86.7% B6, 13.3% CBA; n = 16) as determined SNP genome scanning analysis (Jackson Laboratory) and were maintained by Dr. Simona Chera at UIB before transferring to Dr. Seung Kim at Stanford. Female mice were backcrossed with male B6 CD45.1 for four generations until 96% B6 background and homozygosity for Ptprc^a^ (CD45.1) were achieved (N4). SNP genome scanning analysis was performed after each backcross. Sibling matings with verified heterozygous offspring were used to further breed to RIP-DTR homozygosity after each backcross, a total of four times (F4). In total, mice had been intercrossed for 8 or more generations to achieve the appropriate B6 genetic background and maintain the RIP-DTR transgene for studies here. All animals were housed in non-barrier conditions at the Stanford School of Medicine. Animal experiments were approved by and performed in accordance with the Stanford Administrative Panel on Laboratory Animal Care, accredited by AAALAC International. Experiments were also performed in accordance with ARRIVE guidelines.

### Mouse genotyping

Genotyping for the RIP-DTR transgene was performed using PCR amplification with forward primer (GGT GCT GAA GCT CTT TCT GG) and reverse primer (CTC CTC CTT GTT TGG TGT GG), which produce a 250 bp product. PCR products were analyzed by agarose gel electrophoresis. Dual endpoint qPCR was used for Ptprc^a/b^ (CD45.1/2) genotyping and results reported as Δ normalized reporter value (Rn) with amplification curves shown in Supplementary Fig. S5. DNA was extracted from tail snips and qPCR was performed with TaqMan Gene Expression Assay kit (Thermo Fisher Scientific, Waltham, MA) according to manufacturer instructions, CD45 forward primer (CGC CTA AGC CTA GTT GTG G) and reverse primer (ATT CTT GAT TTT GTT TCC CTA GTG G), as well as CD45.1 Ptprc^a^ (MUT) probe (CCT GAG CCT GCA TCT AAA CCT G) and CD45.2 Ptprc^b^ (WT) probe (CCT GAG CCT GTA TCT AAA CCT GA). To distinguish homozygous RIP-DTR females from heterozygous RIP-DTR females, comparative qPCR was used. DNA was extracted from tail snips above was cleaned using Genomic DNA Clean and Concentrator kit (Zymo Research, Irvine, CA). qPCR was performed with CD45 and RIP-DTR forward and reverse primers used above and SYBR green master mix (Bimake, Houston, TX). Number of copies of RIP-DTR present was calculated by normalizing RIP-DTR cycle threshold (CT) to CD45 cycle threshold to obtain ΔCT, and again normalizing to a homozygous RIP-DTR female confirmed by DT injection to obtain ΔΔCT (data available in Supplementary Table S3. Female homozygotes and heterozygotes exhibited 1 CT difference.

### Flow cytometry analysis of peripheral blood

100 µL of whole blood was collected via the tail vain into EDTA coated tubes. Whole blood was lysed in 1 × RBC Lysis Buffer (Biolegend, San Diego, CA) for 10 min on ice before downstream staining. For analysis of MHC expression, cells were first stained in PBS to distinguish between live and dead cells using LIVE/DEAD Fixable Near-IR Dead Cell Stain Kit (ThermoFisher Scientific). Cells were then incubated with TruStain FcX anti-mouse CD16/32 Fc block (Biolegend) for 10 min on ice in Cell Stain Buffer (Biolegend). Antibodies used for staining from Biolegend were as follows: I-A^k^ PE (10-3.6), I-A^b^ AF647 (KH74), H-2K^k^ FITC (36-7-5), H-2K^b^ PerCP/Cy5.5 (AF6-88.5), CD45.1 PerCP/Cy5.5 (A20), CD45.2 Pacific Blue (104), CD3 FITC (17A2), CD11b BV605 (M1/70), and CD19 PE-Cy7 (6D5). I-E^k^ VioBlue (REA510) was purchased from Miltenyi Biotec (Bergisch Gladbach, Germany). Cells were analyzed with a BD FACSAria II. Data were analyzed using FlowJo software (10.7).

### Induction of diabetes, islet isolation, and transplantation

A one-time 500 ng injection of diphtheria toxin (DT) (Cayman Chemical, Ann Arbor, MI) in Hank’s Buffered Salt Solution (HBS; Caisson Labs, Smithfield, UT) was administered intraperitoneally (i.p.) in adult mice to induce diabetes from β cell ablation. Islet isolation and transplantation were performed as previously described with minor modifications^27,28^. Briefly, pancreases are perfused with 100–125 μg/mL Liberase TL (Roche Diagnostics, Indianapolis, IN) and digested in a 37 °C water bath for 18–22 min. After washing with HBS the crude digest is purified over a discontinuous density gradient, washed once more with HBS, and cultured overnight in RPMI 1640 (Corning; Corning NY) supplemented with 10% FBS, 10 mM HEPES, and 1% penicillin–streptomycin solution. Recipient mice are anesthetized with ketamine and xylazine and given subcutaneous analgesics. After overnight culture, 100–400 islets are loaded into polyethylene (PE)-50 tubing (BD, Franklin Lakes, NJ) and injected under the kidney capsule of recipient mice. After transplant, mice are monitored for wellness daily for up to 10 days. Their body weight and blood glucose (in diabetic animals) are recorded 2–3 × per week using True Metrix Blood Glucose Monitor and Test Strips (Trividia Health, Ft. Lauderdale FL). Diabetic mice are defined as have non-fasting blood glucose > 250 mg/dL. Mice are considered euglycemic when their non-fasting blood glucose returns to < 250 mg/dL. To stabilize blood glucose on the morning of islet transplant, mice received a one-time dose of 40 U/kg of insulin glargine (Sanofi, Bridgewater, NJ) in normal saline. For maintenance insulin in diabetic mice without islet transplantation, 40 U/kg of insulin glargine in normal saline was injected subcutaneously daily for up to two weeks. In diabetic mice maintained for longer than 2 weeks without islet transplantation, subcutaneous Linshin Linbit implants (Toronto, Ontario) were administered monthly to provide continuous insulin release. The number of implants was calculated based on body weight, according to manufacturer’s guidelines. The insulin release is approximately 0.1 U/24 h/implant for 30 days. The nephrectomy procedure involves the same anesthetic regimen as islet transplantation; renal vessels are first tied to prevent hemorrhage and kidney containing islet graft is removed. For hematopoietic cell transplantation, 1.5 × 10^6^ BALB/c CD45.2 hematopoietic cells were transplanted into CD45.1 B6 RIP-DTR recipients to generate mixed chimeras as described elsewhere^29^ with low dose radiation (300 cGy) in place of anti-CD47 prior to transplant.

### Histology

Islet graft and pancreas sections were fixed in 4% paraformaldehyde, embedded in optimal cutting temperature compound, and frozen on dry ice. Embedded grafts were sectioned on a Leica CM3050 S (Leica Biosystems, Buffalo Grove, IL). Standard immunofluorescent staining techniques were used on 6 µm sections. Sections were blocked in 5% BSA for 30 min, incubated with primary antibodies overnight at 4 °C, washed before incubation with secondary antibodies for 2 h at room temperature, and finally washed again. Slide covers were secured with Hard-set Mounting Medium with DAPI (Santa Crus Biotechnology, Dallas TX). Slides were imaged on an EVOS M5000 Cell Imaging System (ThermoFisher) and color channels were merged using Fiji (http://fiji.sc/). Primary antibodies (1:100): αCD3 (17A2) and αCD45 (30-F11) were purchased from BioLegend, insulin (Catalog #: a0564) and somatostatin (Catalog #: A0566) from Dako (Carpinteria, CA), glucagon (Catalog #: PA5-88091) from ThermoFisher. Secondary antibodies (1:1000): CF-594 and CF-488A α-Guinea Pig were purchased from MilliporeSigma (St. Louis, MO); Alexa Fluor-594 α-Rat, Alexa Fluor-488 α-Rat, and Alexa Fluor-594 α-Rabbit were purchased from BioLegend.

## Supplementary Information

Supplementary Information.

## Data Availability

All resources, including B6 RIP-DTR mice, are available from the corresponding author on reasonable request. No datasets were generated or analyzed during the current study.
