# Supplementary material for: Islet cell replacement and transplantation immunology in a mouse strain with inducible diabetes
Source: Sci Rep. 2022 May 31;12:9033. doi: 10.1038/s41598-022-13087-3 (PMC9156753; doi:10.1038/s41598-022-13087-3)
Supplement: Supplementary file 1 — Supplementary Information. [file 41598_2022_13087_MOESM1_ESM.pdf]

Islet cell replacement and transplantation immunology in a mouse strain with inducible diabetes

Preksha Bhagchandani[1]†, Charles A. Chang[1]†, Weichen Zhao[1], Luiza Ghila[2], Pedro L. Herrera[3], Simona Chera[2], Seung K. Kim[1,4,5,6,7]*

[1] Department of Developmental Biology, Stanford University School of Medicine, Stanford, CA, 94305, USA
[2] Department of Clinical Science, University of Bergen, Bergen, Norway
[3] Department of Genetic Medicine and Development, University of Geneva, Geneva, Switzerland
[4] Department of Medicine (Endocrinology Division), Stanford University School of Medicine, Stanford, CA 94305, USA
[5] Department of Pediatrics (Endocrinology Division), Stanford University School of Medicine, Stanford, CA 94305, USA
[6] Stanford Diabetes Research Center, Stanford University School of Medicine, Stanford, CA, 94305, USA
[7] JDRF Center of Excellence, Stanford University School of Medicine, Stanford, CA, 94305, USA
* Corresponding author: Seung K. Kim: seungkim@stanford.edu
† Equal contribution

Supplemental Material

Supplementary Figure S1: Flow cytometry analysis of CBA, B6, and B6 RIP-DTR MHC haplotypes

Supplementary Figure S2: Genotyping for confirmation of RIP-DTR and Ptprc[a]

Supplementary Figure S3: Phenotyping for confirmation of CD45.1

Supplementary Figure S4: Immune cell infiltrate in islet allografts

Supplementary Figure S5: Amplification curves for Ptprc genotyping with dual endpoint qPCR

Supplementary Figure S6: Full size gel for RIP-DTR genotyping

Supplementary Table S1: SNP Genome Scanning Analysis of CBA and B6 Background

Supplementary Table S2: MHC haplotypes for C57BL/6J, CBA/J, and BALB/cJ mice

Supplementary Table S3: Data for comparative qPCR for RIP-DTR genotyping

Fig. S1: Flow cytometry analysis of CBA, B6, and B6 RIP-DTR MHC haplotypes

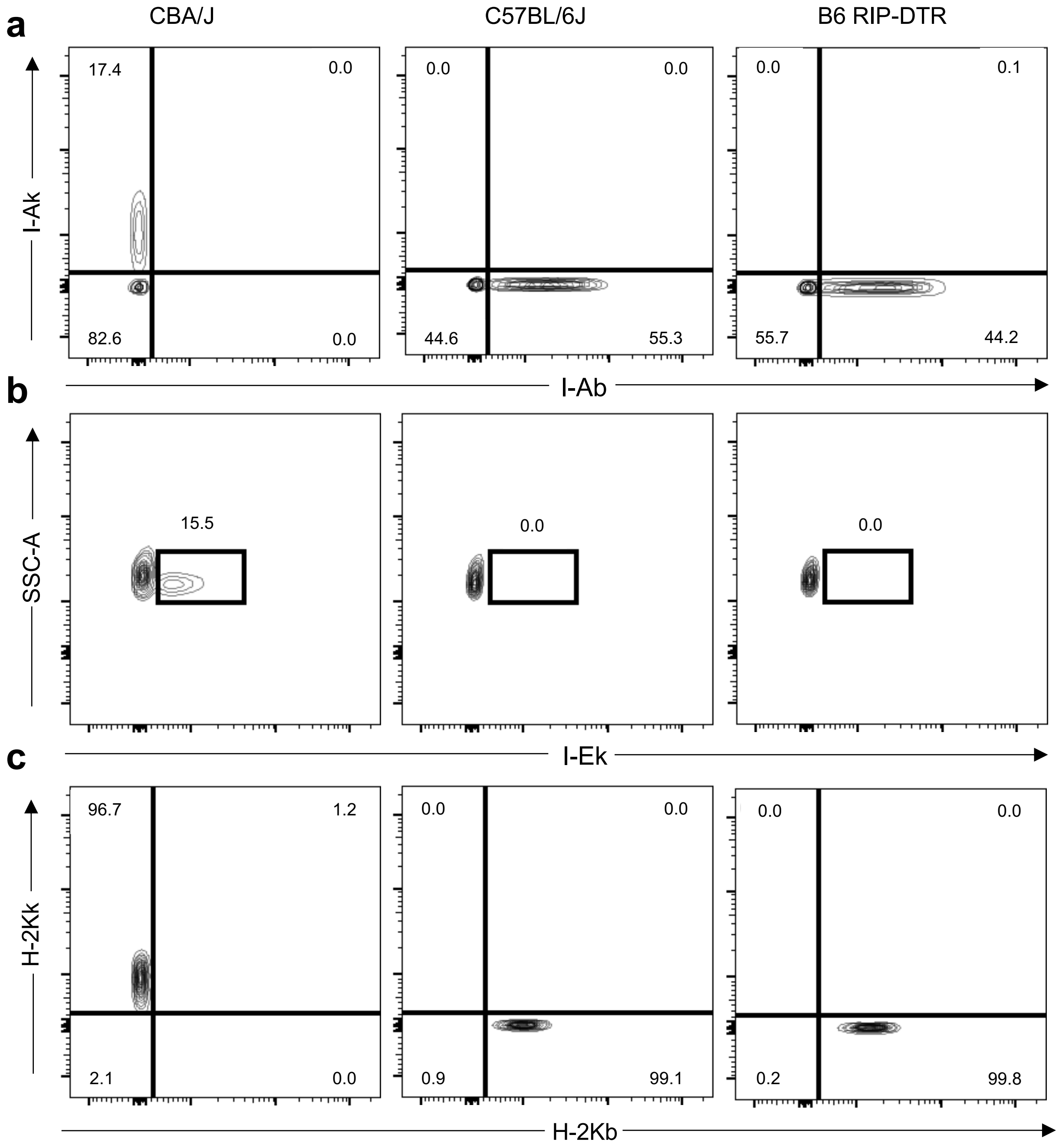

Supplementary Figure S1: A) MHC Class II alleles I-A^b (C57BL/6J haplotype), I-A^k (CBA/J haplotype) and **B)** I-E^k (CBA/J haplotype) analyzed on C57BL/6J and CBA/J controls, as well as B6 RIP-DTR. **C)** MHC Class I alleles H-$2K^b$ (C57BL/6J haplotype), H-$2K^k$ (CBA/J haplotype) analyzed on C57BL/6J and CBA/J controls, as well as B6 RIP-DTR.

Fig. S2: Genotyping for confirmation of RIP-DTR and Ptprc[a]

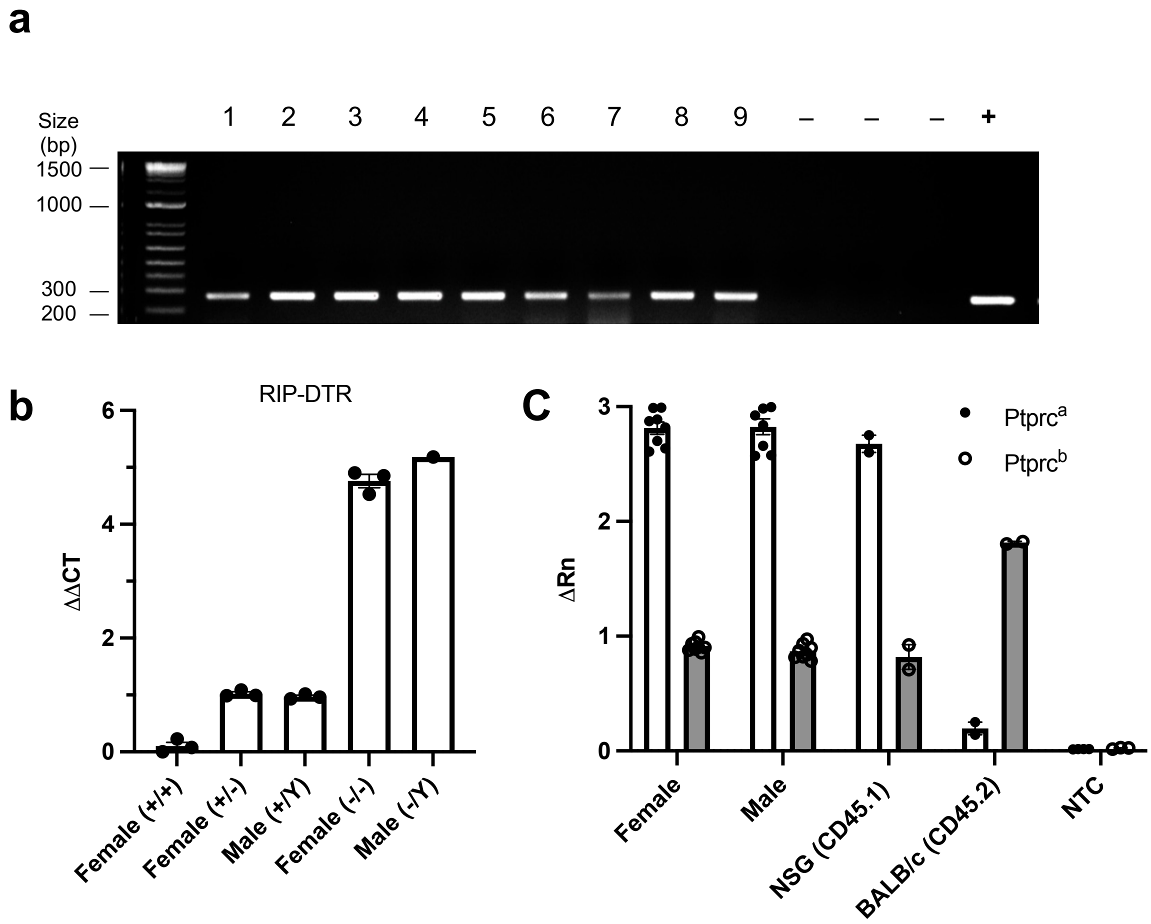

Supplementary Figure S2: **A)** Agarose gel electrophoresis (2% agarose) of PCR amplified products using RIP-DTR specific PCR primers. Lanes 1-4 are RIP-DTR males and 5-9 are RIP-DTR females. Negative control is a B6 mouse. Positive control is a mouse that was confirmed to have RIP-DTR by DT injection. Bp = base pairs. **B)** Comparative qPCR of RIP-DTR normalized to CD45 to determine zygosity of RIP-DTR females, reported as **ΔΔ**CT (mean ± SEM). (+/+) denotes homozygous (n = 3; 0.10 ± 0.07), (+/-) denotes heterozygous (n = 3; 1.02 ± 0.03), and (+/Y) denotes hemizygous for RIP-DTR (n = 3; 0.97 ± 0.02). (-/-) denotes absence of RIP-DTR in females (n = 3; 4.76 ± 0.12). (-/Y) denotes absence of RIP-DTR in males (n = 1). Each circle represents one animal with one replicate. **C)** Dual endpoint qPCR analysis for Ptprc[b] or Ptprc[a] allele showing **Δ**Rn (mean ± SEM) in female (n = 8; Ptprc[a]: 2.81 ± 0.05; Ptprc[b]: 0.91 ± 0.02) and male (n = 8; Ptprc[a]: 2.82 ± 0.07; Ptprc[b]: 0.87 ± 0.02) RIP-DTR mice. CD45. 1 NSG (n = 2; Ptprc[a]: 2.68 ± 0.07; Ptprc[b]: 0.82 ± 0.108) and CD45.2 BALB/c (n = 2; Ptprc[a]: 0.20 ± 0.06; Ptprc[b]: 1.81 ± 0.01) mice were used as controls for Ptprc[a] and Ptprc[b], respectively. Each circle represents one animal with one replicate. NTC = No Template Control (n = 4). Rn = normalized reporter value. CT = cycle threshold.

Fig. S3: Phenotyping for confirmation of CD45.1

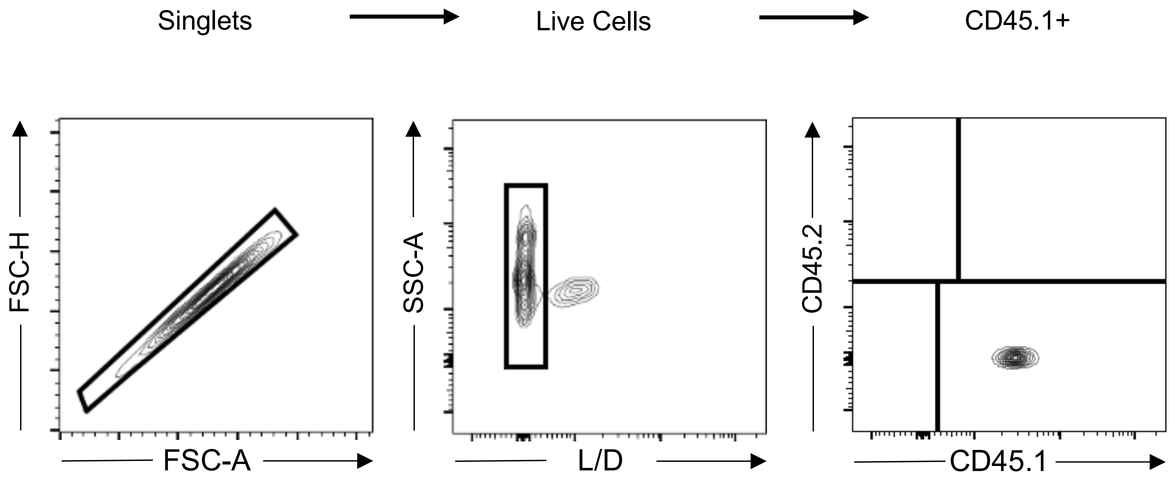

Supplementary Figure S3: Representative flow analysis of immune cells in peripheral blood from naïve B6 RIP-DTR. Live single cells are gated on CD45.1 and CD45.2.

Fig. S4: Immune cell infiltrate in islet allografts

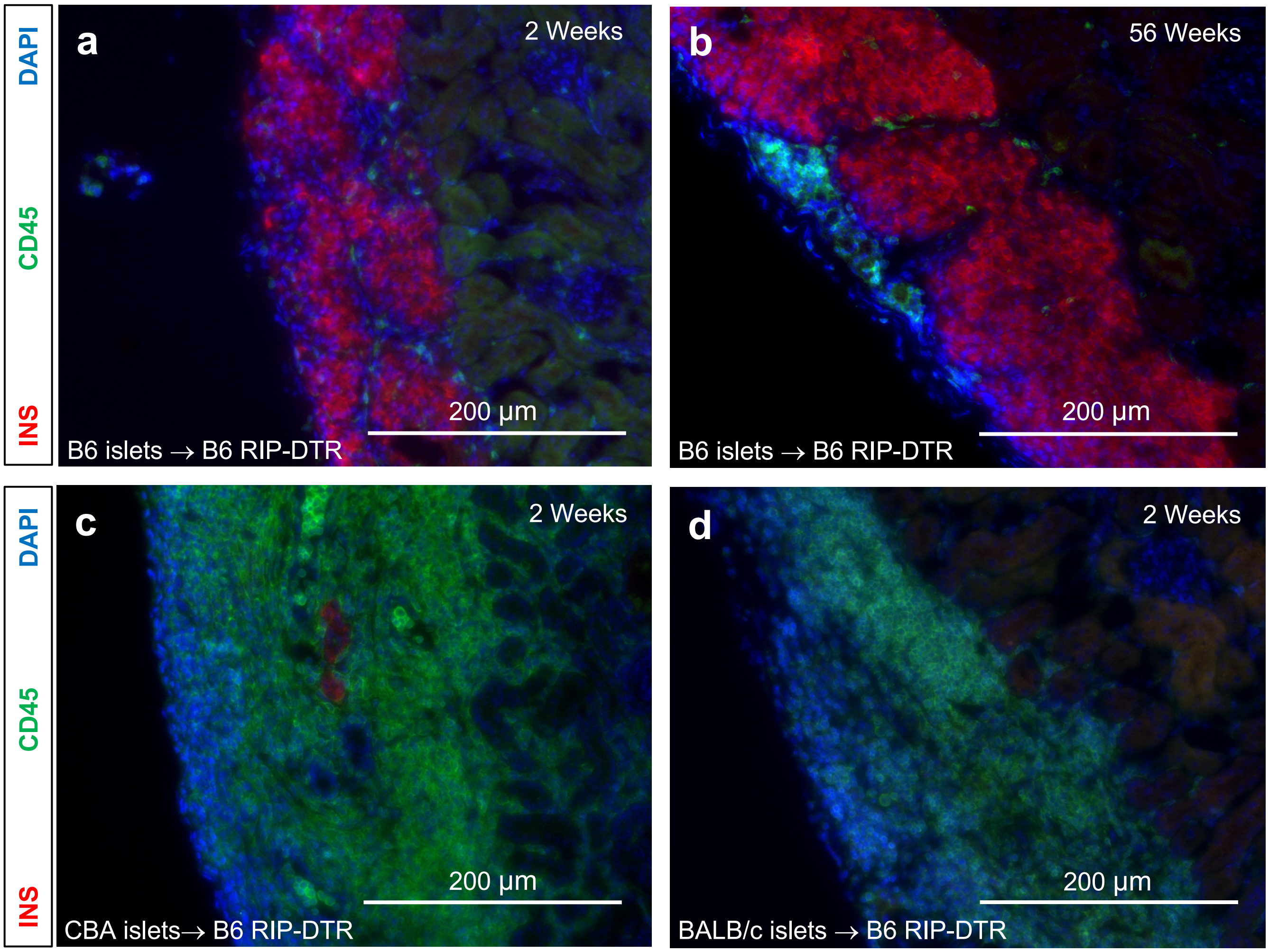

Supplementary Figure S4: A) Representative histology of B6 CD45.1 islet graft at 2 weeks and **B)** 1-year post-transplant into B6 RIP-DTR mice **C)** Representative histology of CBA/J islet graft at approximately 2 weeks post-transplant into B6 RIP-DTR mice. **D)** Representative histology of BALB/c islet graft at approximately 2 weeks post-transplant into B6 RIP-DTR mice. INS = Insulin. CD45 = CD45+ immune cells.

Fig. S5: Amplification curves for Ptprc genotyping with dual endpoint qPCR

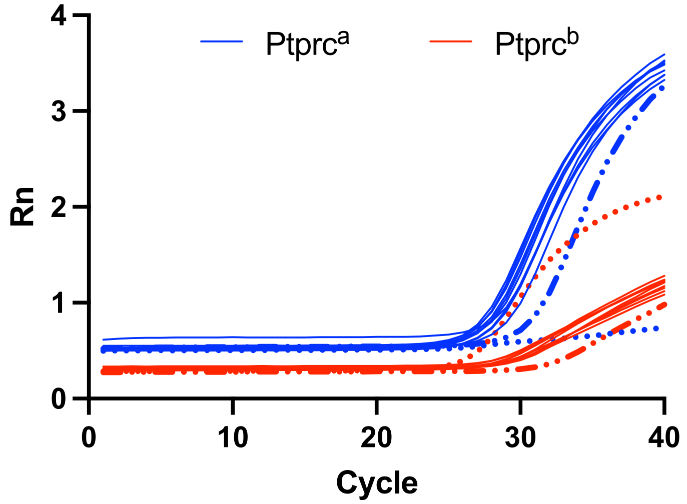

Supplementary Figure S5: Amplification curves for normalized reporter value (Rn) per cycle in B6 RIP-DTR males and females (n=16; solid). Amplification curves for control reference: NSG (CD45.1; dash double dotted) and BALB/c (CD45.2; dotted). Ptprc^a is in blue and Ptprc^b is in red.

Fig. S6: Full size gel for RIP-DTR genotyping

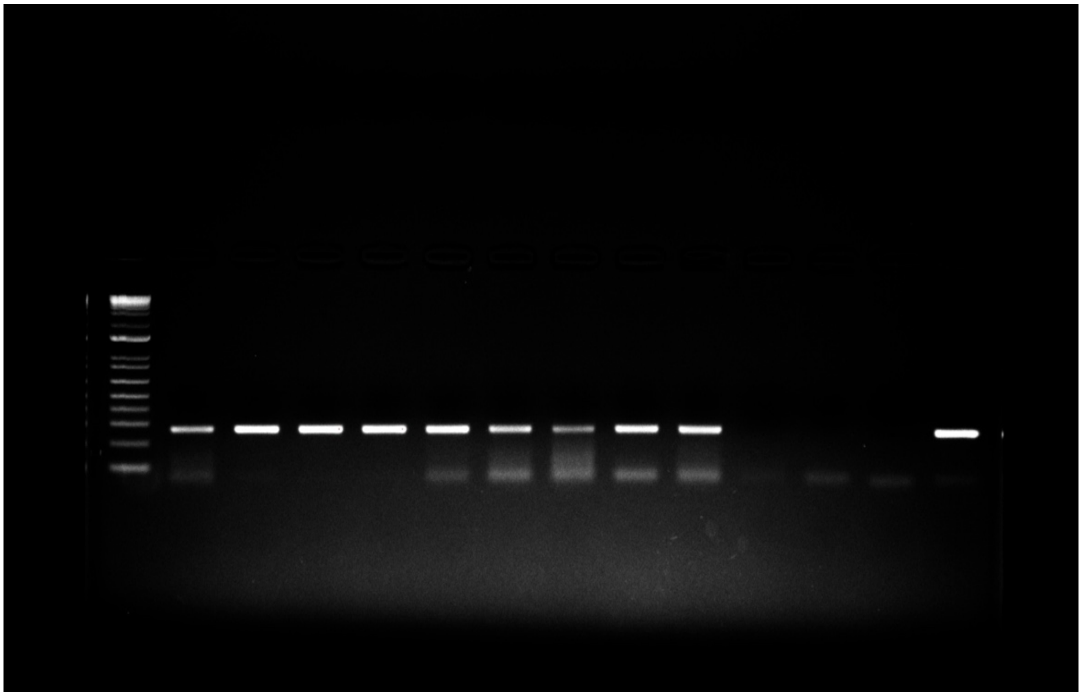

Supplementary Figure S6: Full size gel for RIP-DTR genotyping with 13 lanes. Lanes 1-4 are RIP-DTR males, lanes 5-9 are RIP-DTR females, lanes 9-12 are negative controls (B6), and lane 13 is a positive control (B6 RIP-DTR confirmed by DT injection).

Table S1: SNP Genome Scanning Analysis of CBA and B6 Backgrounds

Chromosome	Number of SNPs assessed	Average Percentage C57BL/6J	Average Percentage CBA/J
1	9	70%	30%
2	9	100%	0%
3	5	92%	8%
4	8	100%	0%
5	6	96%	4%
6	7	100%	0%
7	7	94%	6%
8	6	97%	3%
9	5	100%	0%
10	6	100%	0%
11	6	98%	2%
12	6	93%	7%
13	5	100%	0%
14	5	100%	0%
15	5	100%	0%
16	5	100%	0%
17*	5	100%	0%
18	5	100%	0%
19	4	100%	0%
X	6	84%	16%
**Overall**	**120**	**96%**	**4%**

Supplementary Table S1: Analysis of percentage CBA/J and C57BL/6J background in RIP-DTR mice (n = 12, 6F, 6M) following final backcross to B6 CD45.1 background. *Chromosome 17 contains Major Histocompatibility Complex (MHC) genes. SNP = single nucleotide polymorphism.

Table S2: MHC haplotypes for C57BL/6J, CBA/J, and BALB/cJ mice

		MHC Class I			MHC Class II	
Mouse Strain	MHC Haplotype	H-2K	H-2D	H-2L	I-A	I-E
C57BL/6J	b	b	b	null	b	null
CBA/J	k	k	k	null	k	k
BALB/cJ	d	d	d	d	d	d

Supplementary Table S2: Reference for MHC haplotypes. C57BL/6J, CBA/J, and BALB/cJ mice are all fully MHC-mismatched.

Table S3: Comparative qPCR for RIP-DTR genotyping

Genotype	CT (CD45)	CT (RIP-DTR)
(+/+) REF	18.2610569	22.5383453
(+/+)	17.7893429	22.2944489
(+/+)	16.7465458	21.1019955
(+/-)	15.8669958	21.8348103
(+/-)	14.7563648	20.0272026
(+/-)	15.8669958	21.233532
(+/Y)	15.2960157	20.5377922
(+/Y)	15.5124149	20.8054676
(+/Y)	15.8221626	21.0331707
(-/-)	17.5774517	26.7052059
(-/-)	16.2851734	25.0883121
(-/-)	16.5590115	25.7400169
(-/Y)	15.6953516	25.1541348

Supplementary Table S3: Raw data cycle threshold (CT) values from which **ΔΔ**CT is calculated in Supplementary Fig. S2. Sample marked REF is confirmed homozygous with DT injection and used as a reference for normalization.
